# Protein structure prediction with energy minimization and deep learning approaches

**DOI:** 10.1007/s11047-023-09943-4

**Published:** 2023-05-08

**Authors:** Juan Luis Filgueiras, Daniel Varela, José Santos

**Affiliations:** Department of Computer Science and Information Technologies, CITIC (Centre for Information and Communications Technology Research), University of A Coruña, A Coruña, Spain

**Keywords:** Protein structure prediction, Differential evolution, Evolutionary computing niching methods, Crowding niching method, Deep learning

## Abstract

In this paper we discuss the advantages and problems of two alternatives for ab initio protein structure prediction. On one hand, recent approaches based on deep learning, which have significantly improved prediction results for a wide variety of proteins, are discussed. On the other hand, methods based on protein conformational energy minimization and with different search strategies are analyzed. In this latter case, our methods based on a memetic combination between differential evolution and the fragment replacement technique are included, incorporating also the possibility of niching in the evolutionary search. Different proteins have been used to analyze the pros and cons in both approaches, proposing possibilities of integration of both alternatives.

## Introduction

The structure of proteins largely determines their function, hence the great importance of determining their native three-dimensional structure. For this purpose, traditional laboratory methods, such as X-ray crystallography, Nuclear Magnetic Resonance (NMR) and electron cryo-microscopy, are expensive and time-consuming. As an alternative, computational Protein Structure Prediction (PSP) methods attempt to close the gap between the number of proteins with known sequence (on the order of millions) and the number of resolved proteins with known structure (about 200,000 in the Protein Data Bank - PDB database (http://www.wwpdb.org)).

A first alternative in PSP is the use of templates with structural information of resolved proteins (their structure is known). For example, PSP methods of homology modeling are based on finding a PDB-resolved protein with a homologous amino acid sequence, since with high homology the structures are the same. PSP threading is another possibility if there are no resolved proteins with a homologous sequence. In the latter case, for a target protein and a library of possible templates (folds), threading methods search for the fold in which the target sequence best fits.

In the most difficult and challenging alternative of PSP, called ab initio, only the primary sequence information of the protein (its amino acid sequence) is used. This ab initio prediction is based on Anfinsen’s dogma (Anfinsen 1973), which states that the native structure of the protein is determined solely by the amino acid sequence, as well as that the native structure corresponds to the one with the lowest Gibbs free energy (thermodynamic hypothesis). Consequently, an alternative in ab initio PSP is the use of search methods that attempt to discover the structure with minimum energy, once a protein representation and energy models have been established. The problem is that PSP energy landscapes are high-dimensional and full of local minima. Thus, evolutionary computing search or optimization methods have been used intensively, given their global search capability in multidimensional and multimodal energy landscapes.

This possibility, based on the search for the minimum in the energy landscape, has been one of the traditional PSP approaches, with simplified lattice models for protein representation (Santos and Diéguez 2011; Varela and Santos 2017) and with atomic models (Garza-Fabre et al. 2016), where metaheuristics, especially evolutionary and bio-inspired approaches (Márquez-Chamorro et al. 2015), play an essential role due to the complexity of the search landscape. With atomic models of protein representation, the Rosetta system (http://www.rosettacommons.org) is one of the leading methods for PSP with these energy-based approaches. Rosetta ab initio protocol uses small protein fragments (from resolved proteins) and the classical Metropolis criterion (Metropolis et al. 1953) to decide whether a structural fragment replaces a part of the current conformational structure of the target protein, with the goal of finding the structure with the minimum energy. Working with the fragment replacement technique as a local search operator, our evolutionary/memetic computational solutions (*HybridDE* and *CrowdingDE*) (Varela and Santos 2019, 2020, 2022) outperform Rosetta ab initio protocol when searching for structures with minimal energy and under the same number of conformational energy evaluations.

Nevertheless, a more recent approach in PSP is to predict the contact map or the interdistance map between amino acids, which is a simpler representation of the protein three-dimensional structure. This prediction typically uses the information from Multiple Sequence Alignment (MSA) of the target protein sequence as input to deep learning schemes, such as the initial approaches of *trRosetta* (Yang et al. 2020) and DeepMind’s *AlphaFold* (Evans et al. 2018; Senior et al. 2020). DeepMind’s recent deep learning-based method, called *AlphaFold2* (Jumper et al. 2021), has shown a very large improvement over previous approaches, as demonstrated by the results in the CASP (Critical Assessment of protein Structure Prediction) competition (CASP14 in 2020) (http://predictioncenter.org/). In addition, in a partnership between DeepMind and the European Molecular Biology Laboratory (EMBL), *AlphaFold2* was used to publicly provide structure predictions (over 200 million protein structure predictions including the $\sim$20,000 proteins expressed by the human genome) (https://alphafold.ebi.ac.uk/).

Likewise, *RoseTTAFold* (Baek et al. 2021), also based on a deep learning architecture, has shown a very considerable improvement over the traditional counterpart based on energy minimization, as well as over other deep learning approaches, as tested with proteins in the Continuous Automated Model EvaluatiOn (CAMEO) project (https://cameo3d.org/). Moreover, *RoseTTAFold* allows the prediction of accurate protein-protein complex models only from sequence information, as a new avenue to short-circuit the traditional docking approach.

The objective of this paper is to analyze the advantages and problems of different ab initio PSP strategies (energy minimization and deep learning-based approaches), extending the previous work presented at conference IWINAC 2022 (Filgueiras et al. 2022). The recent methods based on deep learning *AlphaFold2* (Jumper et al. 2021) and *RoseTTAFold* (Baek et al. 2021) were selected, analyzing the problems of these approaches with proteins with few homologous sequences. On the other hand, the widely used Rosetta PSP protocol for the discovery of energy-minimized conformations is also considered, together with our proposals based on memetic approaches between Differential Evolution (Price et al. 2005) and the local search provided by the Rosetta fragment replacement technique.

The rest of the paper is organized as follows. Section 2 provides a brief summary of the main aspects of the PSP approaches considered, while Sect. 3 discusses the comparison of PSP alternatives and their problems using selected proteins. Finally, a discussion based on the results presented is provided in Sect. 4.

## Methods

### Rosetta ab initio protocol

**Fig. 1:**
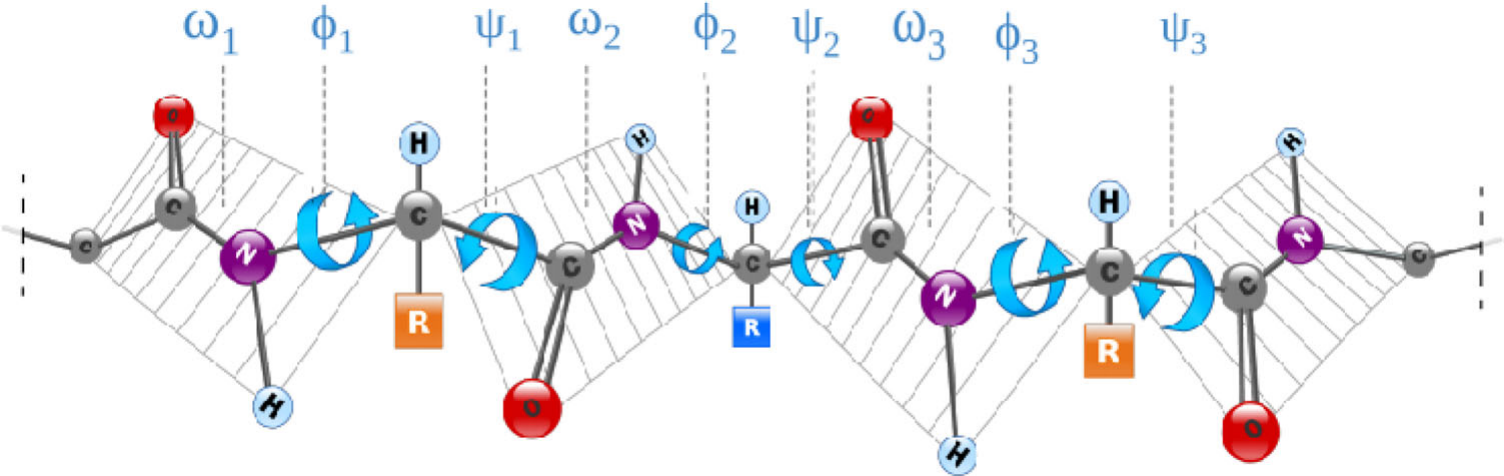
Rosetta’s coarse-grained model for protein representation. It considers only the main atoms of the protein backbone, while pseudo-atoms represent the lateral residues. $\omega$, $\phi$ and $\psi$ dihedral angles encode each protein conformation

Two protein representations are used by the Rosetta system: coarse-grained and all-atom. The coarse-grained representation only considers the main atoms of the protein backbone (with their dihedral angles), whereas the side chains are modeled with a pseudo-atom located at their center of mass (Fig. 1).

The Rosetta ab initio PSP protocol (Rohl et al. 2004) (http://www.rosettacommons.org), with the low-resolution protein representation, employs a search technique in which a Monte Carlo procedure decides whether the dihedral angles of small protein fragments can replace the original ones (Kaufmann et al. 2010; Rohl et al. 2004). A protein fragment is a group of consecutive amino acids of a resolved protein. Fragments are selected by considering their sequence similarity with respect to the window of consecutive residues of the target protein into which the fragments will be inserted. The decision regarding whether the dihedral angles of a selected fragment replace those of the target protein is based on the Metropolis criterion (Metropolis et al. 1953). This criterion always accepts the changes that improve the energy (lower values), while occasionally accepting dihedral angle changes that worsen the energy, with the probability of accepting the fragment depending on the increase in energy relative to the previous state of the target protein.

In Rosetta, the energy of a protein conformation is defined as a weighted linear combination of different energy terms that model the molecular forces acting between the amino acid atoms. For example, steric overlap between backbone and side-chain atoms is penalized, while other Rosetta’s energy terms correspond to van der Waals interactions, electrostatics effects and solvation, hydrogen bonding, repulsion and scores related to protein secondary structure (e.g., helix-strand packing and strand pairing). The detailed definition of the energy terms can be found, for example, in Rohl et al. (2004), and the weight sets for the individual energy terms for the definition of every Rosetta score are detailed in (http://www.rosettacommons.org). The Rosetta score function named *score3* integrates all of the energy components.

**Fig. 2:**
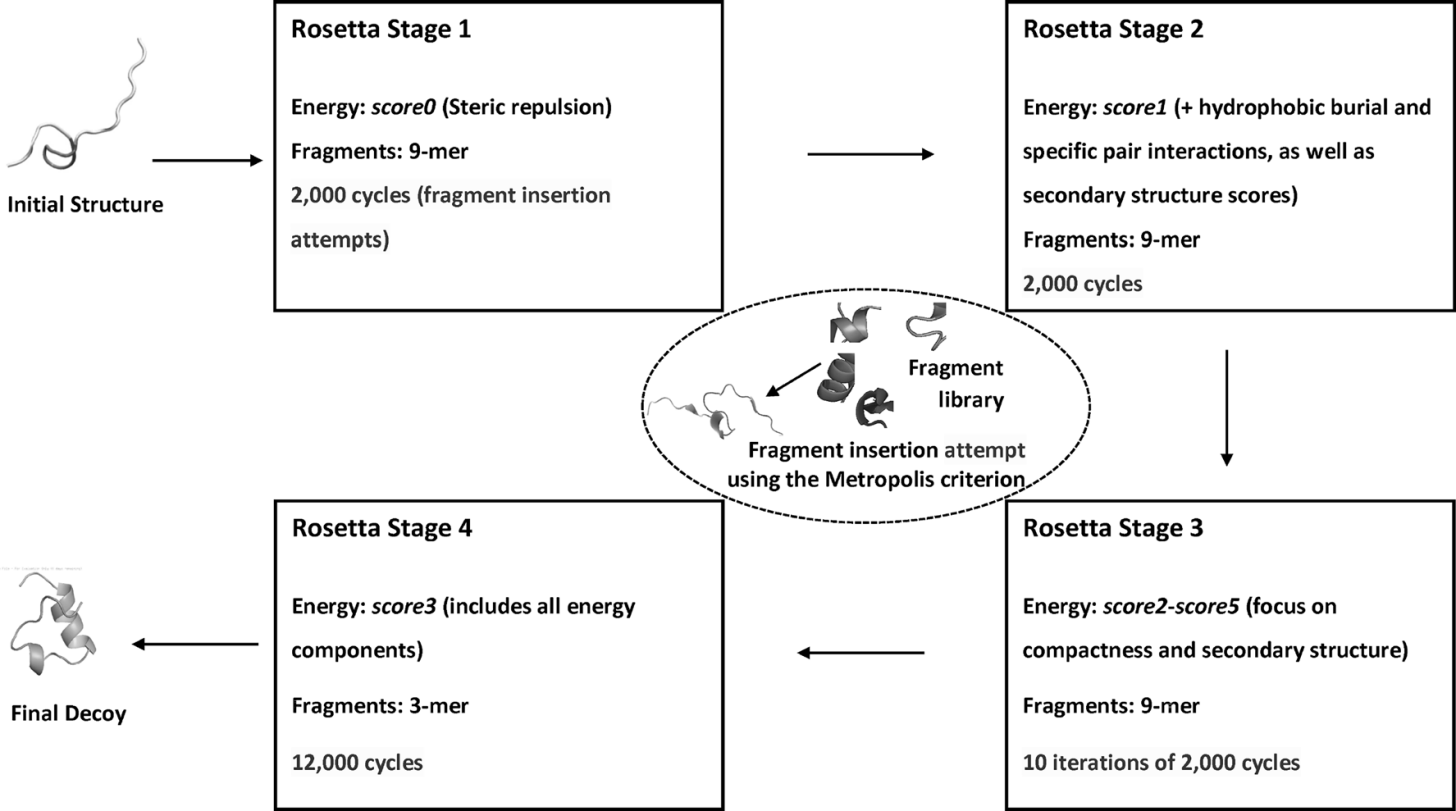
Workflow of the Rosetta Ab initio protocol, working with the coarse-grained representation. Each of the four stages uses a particular score function (incorporating new energy terms in each stage), different number of fragment insertion attempts, as well as fragments with length of 3 or 9 mers

In the search for protein conformations with minimum energy, the stochastic Metropolis Monte Carlo procedure is run thousands of times. For this, the Rosetta ab initio protocol is divided into four stages, which use different score functions (progressively incorporating new energy terms) and number of fragment insertion attempts. Detailed information about these four stages can be found, for example in Rohl et al. (2004) and Varela and Santos (2022), while Fig. 2 illustrates schematically the search process of the Rosetta ab initio protocol with its four stages. Rosetta uses the coarse-grained protein representation and its fragment insertion technique (with the Metropolis criterion) throughout these four stages to generate new structural conformations. The final conformations (“decoys”) in this ab initio protocol, can be refined in an “Ab initio Relax” procedure using the Rosetta’s full atomic model.

### *HybridDE* and *CrowdingDE* PSP approaches

We defined a memetic combination for PSP between Differential Evolution (DE) (Price et al. 2005) and the Rosetta’s fragment replacement technique (*HybridDE* memetic approach). The genetic population encodes possible protein conformations using the Rosetta’s coarse-grained representation (encoding the dihedral angles for each amino acid). The hybrid combination integrates the advantage of the global search of the evolutionary algorithm with the local search provided by the replacement of fragments. The fragment insertion technique is used in DE to refine the population solutions and to refine the DE candidate or trial solutions. The memetic search follows a three-stage evolutionary process, as the fitness of the encoded conformations corresponds to different Rosetta score functions in each stage, while the first stage of Rosetta is used to define the initial population (with partially folded and different conformations). The memetic version is detailed in Varela and Santos (2019, 2020). *HybridDE* outperforms the Rosetta ab initio protocol in obtaining conformations with minimum energy and under the same number of conformational energy evaluations.

**Fig. 3:**
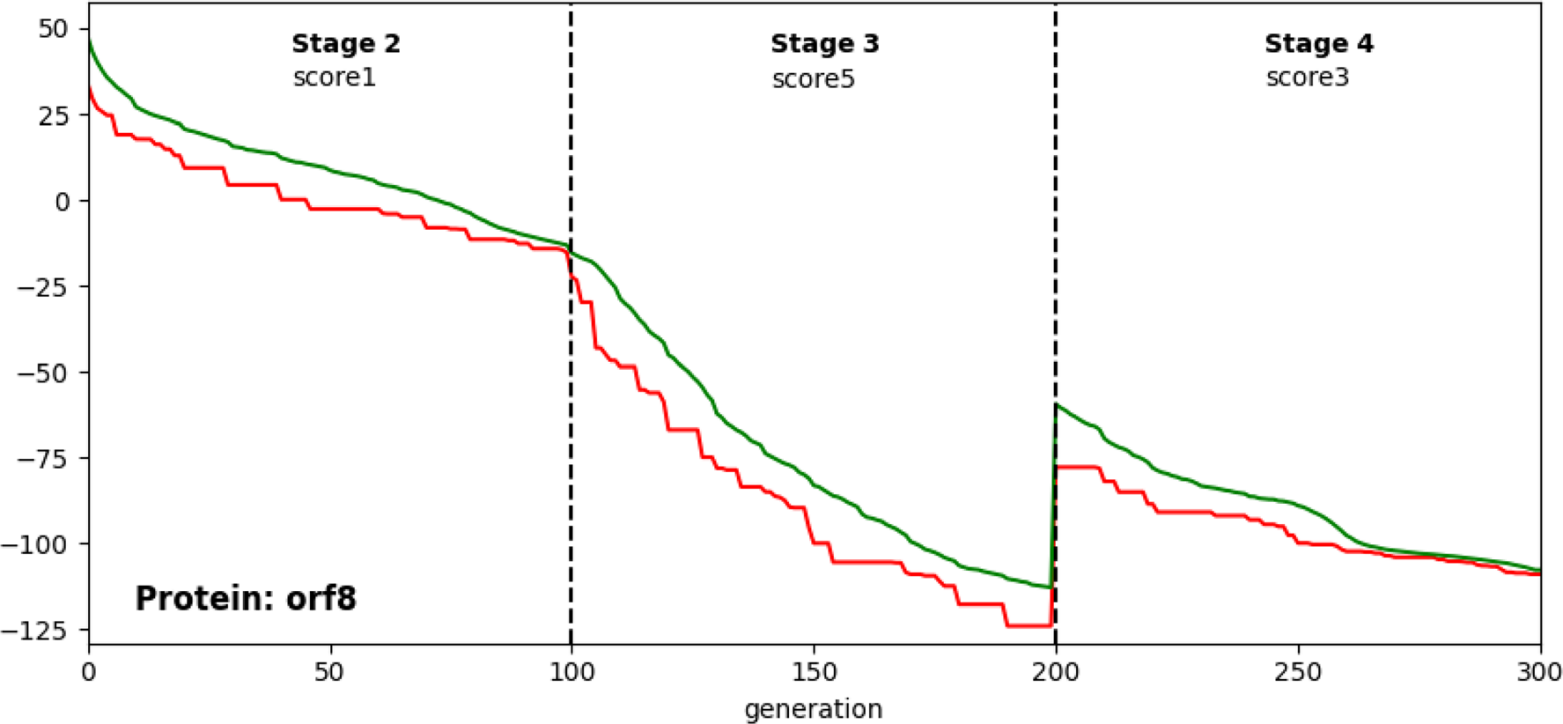
Energy/fitness evolution of *HybridDE* in a run with SARS-CoV-2 protein *orf8* as target: evolution of the average energy of the population (green line) and the energy of the best individual (red line). There are three sequential evolutionary stages, corresponding to the same Rosetta stages (using the same fragment lengths and Rosetta energy score functions)

Figure 3 corresponds to an example of the fitness evolution in the three-stage evolutionary process of *HybridDE*. The example uses an evolution with a population size of 100 individuals, 100 generations (in each of the three evolutionary stages) and SARS-CoV-2 protein *orf8* as target. In each evolutionary phase, the fitness is associated to the energy score that Rosetta uses in its corresponding phase and, consequently, it has different ranges in each evolutionary stage. Note that, using Rosetta nomenclature in Stage 3, *score5* was the final energy (with energy terms focused on compactness and secondary structure), although *score3* in the final Stage 4 is the energy score incorporating all energy components. This evolutionary process with different stages, integrating in each of them more detailed energy functions, allows a progressive structural refinement.

Nevertheless, obtaining energy-optimized conformations is not the only goal when the energy landscape is deceptive (such as the inaccurate Rosetta’s energy model). This occurs when the conformation with the minimum energy does not correspond to the conformation closest to the real native structure. Consequently, one strategy is to obtain a set of optimized conformations (with minimal energy) with structural diversity. For this purpose, we introduced niching methods such as crowding, fitness sharing and speciation into the *HybridDE* version, thus forcing the search algorithm to obtain optimized conformations in different niches (with different structural conformations) (Varela and Santos 2022). This increases the chances of obtaining candidate structures close to the native structure. The crowding niching method was found to be the most useful niching technique in the application, given its simple parameter decision process, defining the *CrowdingDE* version, detailed in Varela and Santos (2020, 2022).

### *AlphaFold* and *RoseTTAFold*

The recent deep learning-based methods of *AlphaFold2*Jumper et al. (2021) and *RoseTTAFold* Baek et al. (2021) for PSP were considered here. *AlphaFold2* is the latest version of DeepMind’s effort in PSP with deep NN architectures, which is an improvement of the first version *AlphaFold* (Evans et al. 2018; Senior et al. 2020). In the case of *AlphaFold2*, the system receives the MSA of a target sequence as input information. MSA algorithms provide the alignment of evolutionarily related protein sequences, with a 2D matrix representation where the horizontal axis represents the residues of the target protein and the vertical axis corresponds to homologous protein sequences with an optimized alignment with respect to the other sequences (alignment that may be suboptimal since heuristics are used).

The idea behind the use of MSA as input information is that correlated mutations between residues indicate their spatial physical interaction. That is, if an amino acid mutates at position *i* in a homologous sequence (with respect to the target sequence), and a correlated mutation appears at position *j* in the same homologous sequence, then it is likely that residues *i* and *j* are in contact in the tertiary structure, since the correlated mutation tends to maintain the protein structure unchanged.

*AlphaFold2*’s architecture is detailed in Jumper et al. (2021). Several self-attention operations are performed in the deep NN architecture. Attention enables the NN to guide the flow of information, by learning to select which aspects of the input information should interact with other aspects of the same input. For instance, MSA representations are processed with consecutive blocks of self-attention in rows and columns. The first generates attention weights for amino acid pairs, allowing identification of which amino acid pairs are most closely related. The second attention process (in the vertical direction of MSA) allows elements belonging to the same target amino acid position to exchange information, i.e., it determines which protein sequences are most informative in the MSA input information.

One of the key modules of the internal architecture of *AlphaFold2* is the main network block called Evoformer, a stack of several NN layers that performs feature embedding. Evoformer works with an embedding of the MSA and with an internal pair representation (a generalized version of a distogram, i.e., a map of interdistances between residues). Both representations exchange information, as updates in the MSA embedding provide new information to change the structural hypothesis in the pair representation, and vice versa.

Evoformer is followed by a NN module or Structure Module that maps the embedding or abstract representation of the Evoformer stack to concrete 3D coordinates of all atoms (as well as the per-residue confidence commented below). In this module, NN attention mechanisms (with invariance to rotations and translations of the protein conformation in space) are used to progressively refine the structure (which includes the side-chain atoms). Finally, the predicted structure information is returned to the Evoformer blocks. Consequently, these two steps (Evorformer and Structural Prediction) are repeated several times in *AlphaFold2* to progressively refine the final and predicted model of the protein.

*AlphaFold2* provides two confidence measures of the predicted structures. The first is the predicted local-Distance Difference Test (plDDT), a per-residue measure of local confidence (on a scale from 0-100). The local-Distance Difference Test (lDDT) is a superposition-free score that evaluates local distance differences of all atoms in a model (Mariani et al. 2013). The plDDT measure provided by *AlphaFold2* estimates how well the prediction would agree with an experimental structure, since it predicts the agreement of the lDDT (lDDT-$C_\alpha$, considering only the atom $C_\alpha$ in each amino acid) between the predicted and real structures. The second metric is the Predicted Alignment Error (PAE). PAE (*x*, *y*) reports the expected position error at residue *x*, when the predicted and real structures are aligned on residue *y*. Consequently, it provides a level of confidence about the relative positions of the amino acids (and different domains) of the protein.

*RoseTTAFold* (Baek et al. 2021) is also a recent method based on deep learning and inspired by the DeepMind’s framework, as the authors state. It is also an improvement over the previous version of the same group at the University of Washington, called *trRosetta* (Yang et al. 2020). *RoseTTAFold* uses a three-track neural network to simultaneously process sequence, distance, and coordinate information. The main new feature in *RoseTTAFold* Baek et al. (2021), with respect to *AlphaFold2*, is the incorporation of a third track in the deep NN design, which operates in the 3D coordinate space. As the authors state (Baek et al. 2021), this provides a tighter connection between the protein sequence, residue-residue distances and their orientations, as well as the coordinates of all atoms. Therefore, the neural architecture has 1D, 2D and 3D tracks with attention mechanisms. There are connections between the three tracks to allow simultaneous learning of relationships within and between sequences, distances and coordinates. *RoseTTAFold* (Baek et al. 2021) outperformed other PSP servers with recent structures submitted to PDB (CAMEO project (https://cameo3d.org/)). *RoseTTAFold* also provides an estimate of the per-residue precision, based on the estimated $C_\alpha$ RMS error, i.e., the predicted distance with respect to the native structure (using only the $C_\alpha$ atoms). This $C_\alpha$ RMS error is estimated from the predicted lDDT-$C_\alpha$, as detailed in Baek et al. (2021).

The *AlphaFold2* (https://colab.research.google.com/github/sokrypton/ColabFold/blob/main/AlphaFold2.ipynb) and *RoseTTAFold* (https://colab.research.google.com/github/sokrypton/ColabFold/blob/main/RoseTTAFold.ipynb) ColabFold servers were used in the predictions. Coupled with Google Colaboratory, ColabFold (Mirdita et al. 2022) is a free and accessible platform for protein folding that integrates the fast homology MMA search of MMseqs2 (Steinegger and Söding 2017) with *AlphaFold2* and *RoseTTAFold*. The commented measures of both deep learning-based methods will be used to analyze the results.

## Results

### Setup of the PSP approaches

The PSP approaches discussed in the Methods section are used with different proteins. In the case of Rosetta ab initio PSP protocol, taking into account its stochasticity, the protocol is run 1,000 times to generate 1,000 decoys (candidate conformations). Rosetta parameter *increase_cycles* is set to 10 (which multiplies the default values of fragment insertion cycles in the different Rosetta stages, details in Rohl et al. (2004) and Varela and Santos (2022)), as recommended on the Rosetta site (http://www.rosettacommons.org).

Regarding the approaches based on DE (*HybridDE* and *CrowdingDE*), the same setup used in Varela and Santos (2019, 2020, 2022) was employed. DE parameters were experimentally adjusted to generate candidate conformations in DE with slight variations with respect to their base individual (random conformation of the population in the DE scheme used), in order to minimize conflicts between atoms in the DE trial or candidate solutions. Therefore, a low weight factor ($F=0.025$) in the mutation operator is needed, along with a high crossover probability ($CR=0.99$). Moreover, DE strategy DE/rand/1/bin was used, which provides low selective pressure (see Varela and Santos (2020, 2022) for details).

With the same purpose of generating 1,000 decoy conformations, *HybridDE* and *CrowdingDE* were run 10 times, with a population of 100 solutions and over 100 generations in the 10 independent runs. Consequently, the 10 runs also generate 1,000 final solutions (joining the final populations of the runs). These DE-based energy minimization approaches will use the same number of fitness/energy evaluations as Rosetta ab initio for generating the 1,000 decoys. It should be noted that energy evaluations are synonymous with fragment insertion attempts, since an insertion attempt involves the energy evaluation of the resulting conformation. The fair comparison is obtained since, in *HybridDE* and *CrowdingDE*, parameter *increase_cycles* is set to 0.1 (100 times less than in the case of Rosetta ab initio protocol), because the evolutionary approaches refine 1,000 conformational solutions with the same Rosetta approach based on fragment insertions, but over 100 generations.

For the deep learning-based approaches (*AlphaFold2* and *RoseTTAFold*), the default configuration provided by the ColabFold servers was used, that is, using the MSA information as input, while the servers provide five candidate models, those with the highest prediction confidence.

### Examples with PDB proteins

Proteins from PDB [20] were selected, i.e., proteins with known structure. Consequently, these proteins serve to test whether the predictions are close to the folded and resolved structure deposited in PDB. Proteins with shallow MSA information (few sequences homologous to the target protein or with low sequence identity) were selected in order to test the behavior of deep learning-based approaches.

**Fig. 4:**
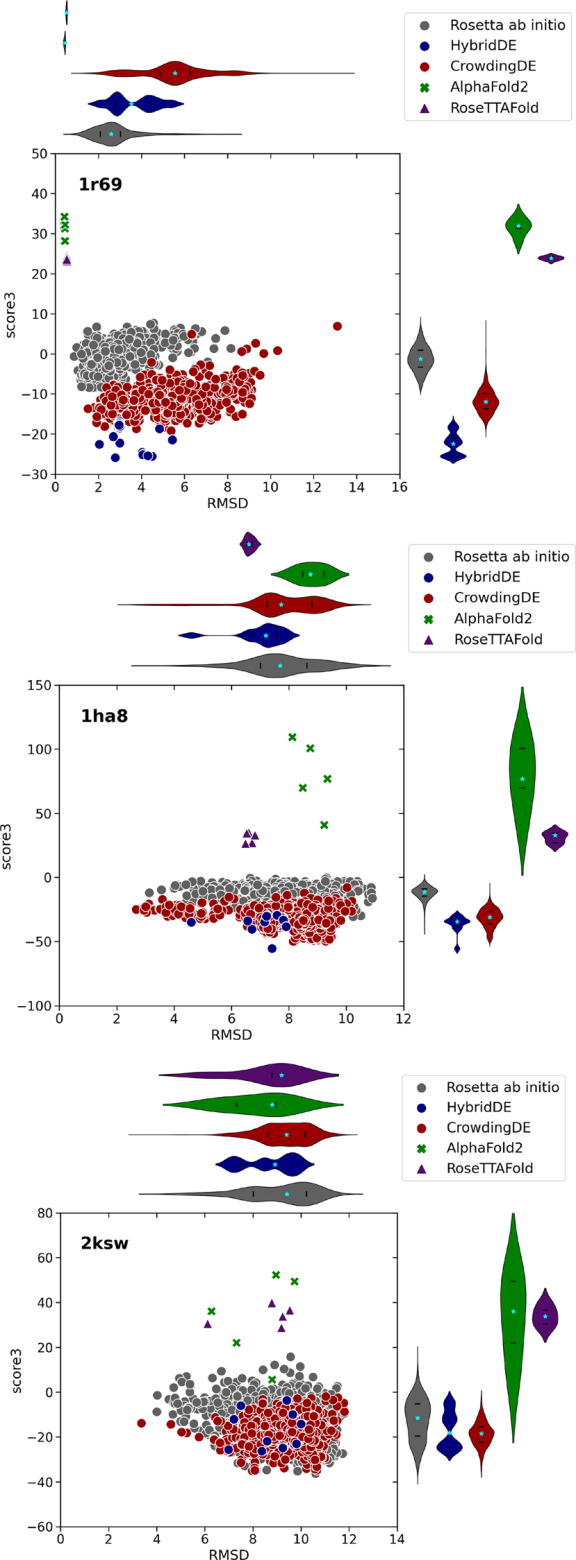
Energy (*score3*) vs. RMSD (from the native structure, in angstroms - Ȧ) for proteins *1r69*, *1ha8* and *2ksw*. Gray: Rosetta ab initio. Blue: *HybridDE*. Red: *CrowdingDE*. Green: *AlphaFold2* solutions. Pink: *RoseTTAFold* solutions. The violin plots correspond with the RMSD (upper) and energy (right) distributions. The quartiles (black lines) and the median (light blue mark) are shown in the violins

A first example is selected in which, even with the scarce information provided by MSA, deep learning methods show excellent prediction results. The example corresponds with protein *1r69* (phage 434 repressor, 69 amino acids). Figure 4 shows the distribution of solutions generated by the different methods. This is a standard graph in PSP for evaluating the performance of energy minimization methods, as it shows the distribution of the optimized protein decoys (in terms of their distances from the native structure), along with the optimization (in terms of energy) obtained in the optimized solutions. The distances of the predicted/optimized conformations from the native structure are calculated with the RMSD (Root Mean Squared Deviation), taking into account the $C_\alpha$ atoms of each amino acid, superimposing each conformation with the native one. The energy of each conformation corresponds to Rosetta’s coarse-grained representation (*score3*, which includes all individual energy terms).

It should be noted that the RMSD distance can only be calculated with proteins whose native structure is known a priori, such as those considered here. That is, with these proteins acting as a benchmark since their native structure is known, we can analyze the quality of the predictions in terms of their structural distance with respect to the native reference structure.

Figure 4 shows that all *AlphaFold2* and *RoseTTAFold* predicted solutions are very accurate, with very low values of RMSD with respect to the native structure deposited in PDB. On the contrary, the energy-based approaches (Rosetta ab initio, *HybridDE* and *CrowdingDE*) present solutions with a large variety of RMSD values, with few decoys with values lower than 2 Ȧ (angstroms) and several solutions with large distances (in RMSD terms) to the native structure.

Nevertheless, in terms of energy of the predicted solutions, the comparison is totally different, since the deep learning-based approaches present solutions with higher energies. This is due to the fact that, in the *AlphaFold2* and *RoseTTAFold* solutions, some atoms have collisions in the side chains, a problem that can be improved by further refinement of the structure. Considering the energy minimization-based approaches, *HybridDE* and *CrowdingDE* obtain solutions with better energy with respect to Rosetta ab initio, showing the better ability of the evolutionary approaches to sample the conformational space under the same number of energy evaluations. However, *HybridDE* obtains the best solution in energy terms, but it corresponds to a solution that is farther away from the native structure with respect to other solutions provided by *HybridDE* and Rosetta. This shows the inaccuracies of the Rosetta energy model, since the solutions with the best energy do not have to correspond to those closest to the native structure, defining a clear deceptive energy landscape for the search algorithms, in which the best approaches in energy terms are those that provide the worst solutions in RMSD terms.

To address the problem of deceptiveness in the energy landscape, the inclusion of crowding in the memetic evolutionary algorithm (*CrowdingDE*), allows us to obtain a wider distribution of optimized solutions, as clearly shown by the distributions in the violins in Fig. 4, but at the cost (in this protein) of presenting solutions not as optimized (in energy terms) as *HybridDE*. Nevertheless, the goal of *CrowdingDE* is precisely this, to present a set of optimized and structurally different proteins, as shown by the distribution. However, even with the inclusion of crowding, in this protein, *CrowdingDE* does not present solutions closer to the native structure with respect to Rosetta ab initio.

**Fig. 5:**
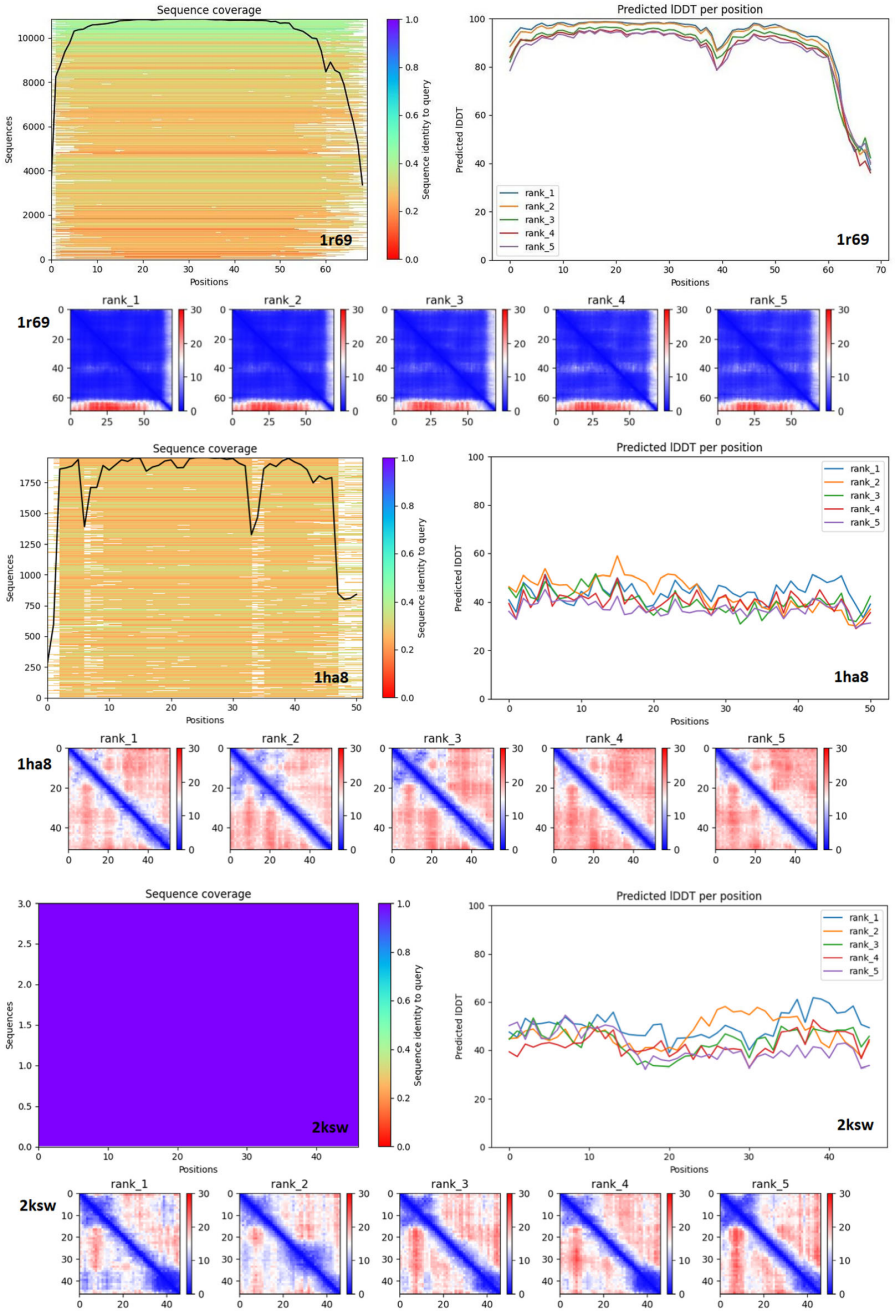
Information about *AlphaFold2* models with proteins *1r69*, *1ha8* and *2ksw*. For each protein, top left: MSA sequence coverage. Top right: predicted Local Distance Difference Test (plDDT) of the predicted *AlphaFold2* models. Bottom figures: PAE (Predicted Aligned Error) of the five highest-rated *AlphaFold2* models

Several comments can be made regarding the *AlphaFold2* and *RoseTTAFold* solutions. Figure 5 shows information about the *AlphaFold2* predictions. Figure 5 includes a representation of the MSA information, which is the same input to *AlphaFold2* and *RoseTTAFold*. The MSA coverage graph shows that there is a large number of homologous sequences, obtained from different genetic databases, although without high sequence identity in most cases. Nevertheless, the solutions provided by *AlphaFold2* present quite high confidence in the predictions and in most of the protein chain. This is shown by the high confidence in the plDDT per residue (explained in Sect. 2.3), where plDDT>90 corresponds to predictions modeled with high accuracy, whereas areas where plDDT<50 should not be considered reliable (Jumper et al. 2021). It is only at the final amino acids that there is a drop in prediction confidence, where there is also worse sequence coverage in the MSA information. Similarly, the PAE graphs for the five best-rated prediction models (considering the average plDDT in the residues) show the high prediction confidence, where the bluer, the lower the estimated error. The PAE graph again shows that only the 10 final amino acids exhibit low confidence in the structural prediction.

**Fig. 6:**
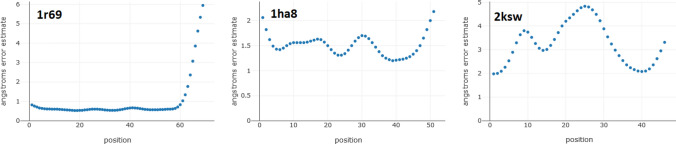
Estimated error ($C_\alpha$ RMS error, in Å) in each amino acid position for the *RoseTTAFold* solutions with the best average confidence and for proteins *1r69*, *1ha8* and *2ksw*

Figure 6 includes the confidence with the RoseTTAFold solutions. The graphs in Fig. 6 correspond to the estimated $C_\alpha$ RMS error (commented in Sect. 2.3) in each amino acid position, that is, the predicted distances (in Ȧ) between the $C_\alpha$ positions (of each amino acid) of the native structure and the predicted *RoseTTAFold* conformational solution. That is, a lower value corresponds to a better prediction, and a zero value corresponds to a perfect prediction of the native structure. For each protein in Fig. 6, its confidence graph corresponds to the *RoseTTAFold* solution with the best confidence (averaged over amino acid positions) of the 5 returned solutions (all five solutions exhibit fairly similar confidence values). The confidence results of the *AlphaFold2* solutions (using plDDT, Fig. 5) and the confidence results of the best *RoseTTAFold* solution (using $C_\alpha$ RMS, Fig. 6) are very similar, with only a drop at the end of the protein chain due to the poor quality of the MSA information at the end of the amino acid sequence.

Regarding computing times, it should be noted that Rosetta ab initio is not parallelized while *HybridDE* and *CrowdingDE* are parallelized in MPI (Message Passage Interface). Typical computing times are 45 min for each of the parallelized 10 independent runs of *HybridDE* and *CrowdingDE* (protein *1r69* as target). Rosetta Ab initio requires an average of 24.64 h to generate 1,000 solutions. The experiments were run in the Supercomputing Center of Galicia (www.cesga.es), with Intel Xeon E5-2680 v3 processors at 2.50GHz and 1GB of RAM. The computing time for the deep learning approaches is variable, since these were run in the ColabFold servers (https://colab.research.google.com/github/sokrypton/ColabFold/blob/main/AlphaFold2.ipynb, https://colab.research.google.com/github/sokrypton/ColabFold/blob/main/RoseTTAFold.ipynb). It can vary from 4 min to 30 min (which includes MSA calculation).

The second example corresponds with protein *1ha8* (pheromone from protozoan E. Raikovi, 51 amino acids). As shown in Fig. 4, with protein *1ha8*, now the energy minimization approaches return solutions closer to the native structure with respect to the deep learning approaches. In the case of the former approaches, *HybridDE* and *CrowdingDE* obtain solutions with better energy with respect to Rosetta ab initio, again showing the enhanced ability of evolutionary approaches to sample the conformational space. Once again, *HybridDE* obtains the best optimized solution in energy terms, a solution that is farther away from the native structure with respect to many other solutions, which again shows the deceptiveness of the Rosetta energy landscape also with this protein. As with the previous example, the inclusion of crowding (*CrowdingDE*), allows us to obtain a wider distribution of optimized solutions. In fact, now *CrowdingDE* presents the closest solution to the native structure.

*AlphaFold2* and *RoseTTAFold* present worse solutions in terms of RMSD distance from the native structure. As shown in Fig. 5, with protein *1ha8*, again the MSA does not present high sequence identity between the target protein and the homologous ones and, in this case, it does not include such a large number of homologous sequences as in the previous protein. This information is not sufficient for fairly high confidence in the predictions. This is shown by the poor confidence in the plDDT per residue. Similarly, the PAE graphs for the five best-rated prediction models show the low prediction confidence. The analysis with the *RoseTTAFold* solutions is similar, as Fig. 6 shows that the confidence (estimated $C_\alpha$ RMS error) is worse with respect to the previous protein and with poor confidence (RMS error greater than 1.5 Ȧ) at both ends of the protein, which is logical as the MSA information also has a drop at the protein ends.

The third example corresponds to protein *2ksw* (a beetle hemolymph protein, 46 amino acids). Figure 4 shows again that *HybridDE* and *CrowdingDE* provide better average energy of the optimized solutions, although Rosetta ab initio also discovers solutions with low energy. As in the previous example, the *AlphaFold2* and *RoseTTAFold* solutions present high RMSD values from the native structure and these are worse with respect to many solutions of the energy minimization approaches. The reason is that, in this case, the MSA map is simple, since there are no homologous sequences found in the genetic databases (except for the sequence itself which was found 3 times). That is, it is an example with no information of proteins with similar sequence. Consequently, the *AlphaFold2* models present very low confidence at all positions in the protein chain, as can be seen in Fig. 5 with the PAE plots and with the plDDT measure.

Similarly, Fig. 6 shows that the confidence of the best *RoseTTAFold* solution is also poor, with estimated $C_\alpha$ RMS errors between 2 and 5 Å. Figure 6 shows that, however, there is no match (considering the amino acid positions with the worst confidence), between the *AlphaFold2* solutions and the *RoseTTAFold* solution (Figs. 5 and 6).

**Fig. 7:**
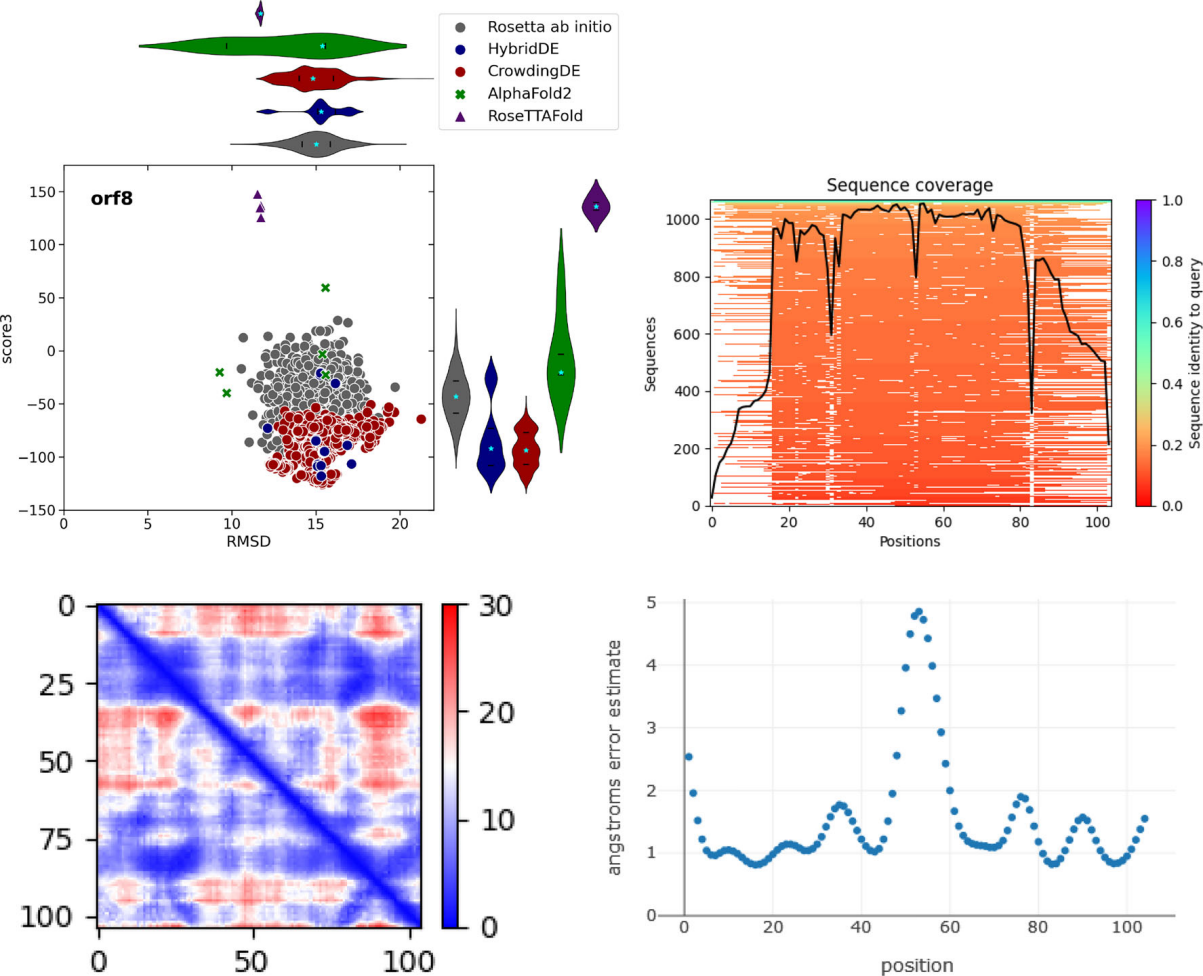
MSA information input to *AlphaFold2* and *RoseTTAFold* with SARS-CoV-2 protein *orf8*. Top left: Energy (*score3*) vs. RMSD with different PSP approaches (same colors as in Fig. 4). Top right: MSA sequence coverage. Bottom left: PAE (Predicted Aligned Error) of the highest rated *AlphaFold2* model. Bottom right: Estimated error ($C_\alpha$ RMS error) per amino acid for the *RoseTTAFold* solution with the best average confidence

The final example corresponds to protein *orf8*, a protein component of the SARS-CoV-2 virus with 104 amino acids (protein visualizations of predictions with SARS-CoV-2 proteins and the prediction methods can be seen in (https://www.dc.fi.udc.es/ir/in845d-02/SARS-CoV-2_protein_prediction/index.html)). This protein has no homologous proteins in the PDB database [20]. Even with the search for homologous sequences in genetic databases, the MSA coverage is poor (Fig. 7, top right). Consequently, the prediction confidence of *AlphaFold2* and *RoseTTAFold* models is low.

Neither approach presents accurate solutions in RMSD terms, as shown by the distribution of solutions in the energy vs. RMSA plot (Fig. 7, top left), showing the difficulty of some proteins for the different PSP approaches. Clearly, the solutions with deep learning approaches need a posterior refinement, especially in the case of the *RoseTTAFold* solutions. Finally, the prediction confidence of these latter approaches shows that the best *RoseTTAFold* solution and the best *AlphaFold2* solution exhibit low confidence at the ends and middle positions of the protein, especially the *RoseTTAFold* solution at the middle amino acids.

## Discussion and conclusions

This study has performed a comparison between PSP approaches based on energy minimization and deep learning. It is clear that energy minimization approaches present better solutions in terms of minimized energy with respect to deep learning approaches. In addition, in energy minimization-based alternatives, memetic approaches show better sampling of the energy landscape with respect to the state-of-the-art Rosetta ab initio protocol. However, imperfections in the energy landscape do not allow the best optimized solutions with memetic approaches to correspond to solutions closer to the native structure. The main conclusions on the comparison of the approaches considered can be summarized in Table 1.

**Table 1:** Summary of the main conclusions that can be drawn from the comparison of the results with the different protein structure prediction approaches considered

Approaches based on energy minimization obtain better energy-optimized conformations than those based on deep learning.	
In the energy minimization-based approaches, the memetic approaches (*HybridDE* and *CrowdingDE*) better sample the energy landscape with respect to Rosetta ab initio under the same number of energy evaluations/fragment insertion attempts.	
The incorporation of the crowding niching method into the memetic algorithm (*CrowdingDE*) allows obtaining a set of optimized conformations with a higher structural diversity with respect to *HybridDE*, which is useful in proteins with a deceptive energy landscape.	
Deep learning-based approaches (*AlphaFold2* and *RoseTTAFold*) provide better solutions, in terms of distance (RMSD) from the native structure, for most proteins (with known structure). However, their predictions are not reliable when MSA information is poor.	
If the input MSA provides sufficient information, deep learning-based approaches tend to provide high quality predictions of the protein backbone, while the side chains tend to present collisions. A refinement process, based on energy minimization, can improve their initial predictions.	

Predictions with selected proteins show the dependence of deep NN-based approaches on MSA input information. When the MSA information is not detailed enough, deep NN-based approaches can present predictions with low confidence, as shown with selected proteins. As also stated by Peng et al. Peng et al. (2022), there are proteins in which deep learning based methods relying on MSA information present poor predictions, such as proteins from viruses without homologous sequences in genetic databases, which is the case of the last example with a protein of the SARS-CoV-2 virus. In this sense, a work to be done will be an analysis of the correlation between the quality of the MSA information and the confidence of the prediction using a large number of proteins.

Nevertheless, it must be taken into account that proteins with low sequence identity were chosen for the analysis, and the recent approaches based on deep learning present predicted solutions with very low distances to the real native structure in the vast majority of proteins, showing a great leap forward in this problem of computational structural biology. For example, in the case of the human proteome, 58% of residues present a confident prediction (pLDDT> 70) with *AlphaFold2* Tunyasuvunakool et al. (2021).

However, deep learning-based methods cannot provide different predicted structures for proteins mutated in a few amino acids, as discussed in Buel and Walters (2022); Callaway (2022); Peng et al. (2022). Moreover, predictions with deep learning schemes present solutions with high energy, in most cases due to conflicts between atoms in the side chains. In all cases, a refinement process is needed to resolve these conflicts. A refinement process that integrates the different approaches is a line of research to be explored, using evolutionary algorithms to refine (in energy terms) the predicted conformations. The initial population may include different prediction models of different PSP alternatives, so that evolutionary optimization can obtain a consensus refined model of the PSP approaches, thus integrating the different approaches.

## Data Availability

The data that support the findings of this study are available from the corresponding author upon reasonable request.
